# A Just Transition for Antimicrobial Resistance: The Need for More Forms and a Broader Scope of Justice

**DOI:** 10.1017/pub.2025.10062

**Published:** 2025-10-17

**Authors:** Natalie Tegama, Lovro Savić, Susan Bull, Caesar Atuire, Tess Johnson

**Affiliations:** 1 Centre for Tropical Medicine and Global Health, University of Oxford, Oxford, UK; 2 Ethox Centre, Oxford Population Health, University of Oxford, Oxford, UK; 3 Faculty of Medical and Health Sciences, University of Auckland, Auckland, New Zealand; 4 Pandemic Sciences Institute, Nuffield Department of Medicine, University of Oxford, Oxford, UK

**Keywords:** antibiotic stewardship, antimicrobial resistance, climate policy, epistemic justice, just transition

## Abstract

Antimicrobial resistance (AMR) is a global public health challenge that, like climate change, demands urgent, coordinated, multi-sectoral action. Yet, responses to AMR may be ill-suited to local contexts, overlook historical inequalities, or dismiss marginalised knowledge systems. Some of these concerns can be discussed using the concept of a just transition, which aims to ensure that “no one is left behind,” “all voices are heard,” and past injustices are addressed. However, framing justice in these terms is insufficient. We argue for a more multifaceted and broader-scoped understanding of what justice demands in a just transition for AMR. We examine existing justice frameworks in AMR literature and discuss two cases that motivate our call for including both more forms of justice in a multifaceted concept of a just transition and a broader scope of justice. The first case involves over-the-counter antibiotic access in the Kibera informal settlement near Nairobi, highlighting structural injustices resulting from colonial oppression and what an Ubuntu philosophy would show as injustice. The second case concerns veterinary prescription requirements for Maasai pastoralists’ livestock farming in southern Kenya and highlights epistemic and distributive injustices, as well as injustices that befall non-human animals. These examples reveal distinct injustices shaped by socio-cultural and ecological contexts.

## 1 An introduction to antimicrobial resistance ethics

Antimicrobial resistance (AMR) involves the evolution of microbes in response to their environments, so that they can still reproduce and aren’t killed by antimicrobials (including antibiotics, antivirals, and antifungals), whether naturally occurring or manufactured. Foci on addressing AMR are generally split across innovation efforts to produce new antimicrobials, and antimicrobial stewardship efforts, which aim to protect the existing stock of effective antimicrobials by curbing “unnecessary,” “indiscriminate,” or “irrational” uses of antibiotics.^1^ As a public health challenge, AMR is a “superwicked” problem requiring international, multi-sectoral coordination against outbreaks of multiple resistant microbes that can affect animal health, human health, and the environment.^2^ In this respect, it is similar to the superwicked problem of climate change.

AMR is also similar to climate change in that efforts to address it, if not implemented with proper consultation and appropriately, risk exacerbating injustices. Actions can be poorly adapted to specific contexts, failing to consider historical contributions to the problem or account for important sources of knowledge. To remedy this within the context of climate change, the concept of a *just transition* has been applied.^3^ A just transition is traditionally held to involve moving to an environmentally sustainable future through changing to green energy sources in such a way that burdens and benefits are distributed fairly, “no one is left behind,” “all voices are heard,” and past injustices are compensated.^4^ It can also be applied to the AMR context, to involve transitioning to a future relationship with microbes and antimicrobials in a way that leaves no one group unfairly worse off (distributive justice), hears all voices (epistemic/procedural justice), and compensates for injustices in current or historical access to or reliance on antimicrobials resulting from systemic inequities (restorative justice).^5^

Yet, the concept of a just transition is somewhat limited as it stands. The questions and scope of justice that particular instances of AMR disease or stewardship actions raise clearly go beyond distributive, procedural, restorative, and even the less-often-recognised epistemic justice. They involve the way communities are treated and their very identity and meaning for members, structural pressures that perpetuate reliance on antimicrobials, links in injustices borne by humans, animals, and the environment, and more. In this paper, our main aim is to explore why we might need a multifaceted and broader approach to what “justice” requires for a “just transition” in the AMR context. We advocate for broadening both the forms of justice included within a just transition and the scope of application of justice (across human and non-human subjects, and across space and time).^6^ Admittedly, while our paper provides sufficiently compelling reasons to incorporate under-recognised but important ideas of justice in the context of AMR, our attempt to provide a *full* and *comprehensive* account of a “just transition” remains incomplete in the sense that we refrain from engaging with or explicating the idea of “transition.” Nevertheless, as this paper demonstrates, focusing on one constitutive part of the concept of “just transition” (i.e., “justice”) provides the first significant step in illuminating the idea that our responses to the global public health challenge of AMR require a different conception of a just transition. Another limit in the scope of our work in this paper is our focus on AMR stewardship interventions, as opposed to drug innovation. This is because stewardship interventions can typically raise more significant justice-related questions.

To start, we present some background (Section 2) on the idea of a just transition and the sociopolitical context in which the problem of AMR has arisen (or, perhaps, the context that has created it). Next, we touch upon existing ethics literature on AMR with regard to justice, and discuss what work remains to be done to build a more holistic picture of what a just transition entails (Section 3). We go on to examine two particular case studies of stewardship measures in the Kenyan *National Action Plan on Prevention and Containment of Antimicrobial Resistance* (NAP), which affect populations, including residents of the Kibera slum on the outskirts of Nairobi, and the Maasai people living as pastoralists in southern Kenya.^7^ We demonstrate how enforcement of bans on over-the-counter (OTC) access to antimicrobials, along with requirements for withdrawal periods and veterinarian prescriptions for animal antibiotic use, may raise issues of justice. Without proper adaptation or tailoring to the needs of communities, these measures may fit into what we intuitively consider an *unjust transition*. These case studies allow us to highlight why a broad-scope, multifaceted concept of a just transition is needed (Section 4).

## 2 Background on justice transitions

Historically, the just transition movement grew out of labour union action in the 1970s, which demanded fair compensation for those exposed to harmful pollutants through their work, and for those whose jobs were made redundant through coal mine closures.^8^ Since then, it has been put to various purposes, including as a means of developing an integrated framework for energy justice, as a theory for socio-technical transitions more broadly, as a strategy for governance, and as an interpretation of public perception.^9^ The emphasis primarily on pragmatic development and use of the concept of a *just transition*—across all these purposes—has kept the forms and scope of justice included within the whole concept of a just transition relatively narrow. The idea of “leaving no one behind” by bridging skills gaps for workers previously reliant on the fossil fuel industry can refer to the requirements of distributive justice, certainly. In parallel, “hearing all voices” by integrating methods of public participation or deliberation into policymaking on climate mitigation can refer to procedural justice. Alternatively, or in addition, it can refer to ensuring that often-marginalised knowledge systems are respected and valued, including in understanding traditional approaches to climate mitigation.^10^ And “compensating past harms” through payments to those affected by workplace-associated chemical exposures and resulting health issues can refer to restorative justice. These forms of justice are certainly discussed in the core just transition literature.^11^ However, this limited set fails to highlight other intuitive requirements for treating people, animals, and the environment in a just way, and therefore it is inadequate for avoiding an unjust transition.

The concept of a *just transition* is now being applied to the problem of AMR.^12^ There is much room and need for a concept that can help us to avoid injustice in efforts to mitigate AMR. We must ensure not only that those who have grown reliant on antimicrobials in navigating their everyday lives or their work aren’t left behind, and that the voices of historically marginalised groups are heard, and that procedures are fair, but also that considerations of how we live within ecosystems and how we interact with animals, plants, and microbes are included in our approach. We need to consider the structures and sociopolitical contexts that have led to both severe health outcomes from AMR and community-level reliance on antimicrobials as part of everyday life. To achieve a balanced and fair approach in our mitigation measures, a broad range of justice forms must be fulfilled across a scope that is global, applies to animals and the environment, as well as humans, and considers past harms and future moral subjects.

Access to underlying determinants of health, including clean water, safe food, sanitation, and housing, has been recognised as foundational for human health.^13^ The UN Sustainable Development Goals have been hailed as an unprecedented opportunity to resolve the dilemma between investing in the social and economic determinants of health and investing in healthcare systems alone.^14^ While substantial efforts to improve access to social and economic determinants of health have been achieved, significant and entrenched structured inequities—that is, those mediated by formal institutions—remain, and many populations continue to be adversely affected by multiple, intersecting social determinants of health, which result in increased health risks from infectious disease and AMR, as well as complex barriers to the use of health services.^15^

Against a context of inequitable access to clean water, safe food, sanitation, and housing, the rapid transmission of antibiotic resistance genes and organisms among people, animals, and environments (air, water, soil, and food) is well documented.^16^ Our reliance on, and massive use of, antimicrobials—including to address the impacts of both structured and structural inequities—and the resulting spread of AMR are profoundly influenced by a broad range of biological, social, political, and economic drivers in heterogeneous neoliberal health landscapes. These are further ingrained via formal institutions that shape access and behaviours. Dominant discourses centring on the misuse and overuse of antimicrobials in humans, animals, and plants as primary drivers of AMR drive policy responses seeking to control access to antimicrobials, rather than addressing structured and structural inequities in exposure to drug-resistant infections and antimicrobial pollution, and the social, political, and economic priorities driving demand.^17^

## 3 Justice in existing ethical analyses of antimicrobial resistance

Justice is not a new idea in the ethical analysis of AMR and stewardship measures, but the forms of justice that have been explored in the literature to date—although broader than the conceptions of justice included in the concept of just transition—are still somewhat limited. We have informally surveyed the existing AMR ethics literature and found that it focuses primarily on forms of justice, including distributive, procedural, and structural justice, with much less or no focus on other forms, such as epistemic justice, more-than-human justice, Ubuntu justice, justice as *vincularidad*, Confucian justice, or Buddhist justice.^18^ It is also necessary to highlight that the *scope* of justice should be expanded as we consider moral subjects beyond humans, highlighted by more-than-human or multi-species justice.^19^ The result of this limitation is that there may be issues of justice arising in the AMR context that aren’t captured by the forms or scope of justice usually discussed. For instance, we may need one of these forms of justice in a conception of what a just transition requires in order to articulate the moral duties raised by the bonds between beings: *Vincularidad* is the awareness of the relationships between all living organisms, and with land and space. Justice in the form of *vincularidad* attends to the bonds between beings and the moral duties this raises.

The most widely cited bioethics article that included the topic of AMR and distributive justice focused on justice as one among a range of ethical challenges arising from AMR, ranging from those arising in the context of drug and diagnostic tools development, over ethical problems in relation to agricultural and farming practices, to ethical obligations towards future generations.^20^ Treating AMR as a problem of allocating antimicrobial resources, the question is ultimately limited to how these resources ought to be shared out, an approach that has been mirrored in other papers.^21^

Another approach in the AMR ethics literature primarily centres around procedural justice. Most explicitly in relation to procedural justice, Charani et al. hold that the Global Action Plan on AMR failed to ensure that countries recognise inequalities in their populations’ vulnerability to AMR, or that in designing individual National Action Plans, diverse populations are included in deliberative processes.^22^ Work on procedural justice highlights the importance of deliberative processes for the formulation of policy on AMR that incorporate the views of disadvantaged populations.^23^ There is room for expansion here in the area of positive recommendations on how to improve the space of public deliberation and how public deliberation could incorporate the views of disadvantaged populations, linking in with possibilities to promote epistemic justice.

Epistemic injustice refers to instances in which a person is “wronged specifically in their capacity as a knower.”^24^ In the context of AMR, epistemic injustice might come to the fore in cases where different types and sources of knowledge are either inequitably valued or fully devalued.^25^ Tegama’s work explicates on-the-ground data that reflect ways in which healthcare workers (as experts) learn to tackle AMR.^26^ This literature is small and presents an opportunity for development to assess whose knowledge about AMR is respected, and who has access to this knowledge.

Relatedly, there is a small but growing area in ethics of AMR literature that seems to reflect an increasing interest in AMR and its relation to a specifically “structural” conception of justice. Interestingly, the scope of justice is also often broader in these literatures, applying to moral subjects globally. Structural injustice is “when social processes put large groups of persons under systematic threat of domination or deprivation of the means to develop and exercise their capacities, at the same time that these processes enable others to dominate or to have a wide range of opportunities for developing and exercising capacities available to them.”^27^ In the AMR context, Merrett et al. briefly discuss improving diagnostics, access, and governance in the context of social inequalities caused by underlying processes and contextual factors. Krockow and Tarrant focus more on structured justice, discussing antimicrobial stewardship challenges as differing across national contexts around the globe, particularly according to economic status and healthcare system type.^28^ These institutions have a significant impact on how burdens and benefits of antimicrobial use are distributed and who is recognised as a legitimate user of antimicrobials. Secondarily, the literature links development narratives to justifications for stewardship measures, calling for context-specificity in ethical justifications for such measures on the basis of structured and structural considerations, but more work is needed.^29^ This may be a key form of justice for integration into a multifaceted concept of a just transition.

Finally, bringing in the form of restorative justice alongside structural, structured, and social justice, Reid discusses the imposition of social development narratives in the AMR context and the compounding of injustices regarding which populations have historically had access to antimicrobials (mostly colonial powers), which countries are currently suffering the greatest impact from resistant diseases—mostly low- and middle-income countries (LMICs), and which countries’ populations are bearing the most blame for “inappropriate” antibiotic use.^30^ Reducing child mortality from infectious diseases is a case in point. While successes of this sort are standardly attributed to the development in political economies, the growth in gross domestic product (GDP) that subsequently resulted in urbanisation in high-income countries has not been re-distributed globally in a way that improves public health infrastructure and living standards for all. According to Reid, the main reason for this rests on the fact that political empowerment necessary for these successes is limited in LMICs and that these limitations are imposed by the actions of global corporations that “bring (some) growth in income in LMICs.”^31^

On the whole, dominant justice concerns in the bioethics literature on AMR centre around the distribution of antimicrobial resources, critiquing processes that fail to consult the right stakeholders, and considering existing structural and social disadvantages that might affect the fairness of stewardship policies. These forms of justice are primarily based in the Western philosophical tradition, and tend as a result to centre on individualistic notions of what a person is owed, and to centre on the distribution of harms and benefits. Whilst this literature on AMR and justice is useful, a more robust approach may be to use many forms of justice and a wider scope, in order to see issues through many different lenses, to see how a just transition can be achieved, and to see who should count as a subject of justice—including non-humans—as illustrated below.

## 4 Case studies of antimicrobial stewardship measures

We take an approach that acknowledges the importance of multiple forms and a broad scope for justice in ethically assessing considerations relevant to antimicrobial stewardship measures that might form part of a just transition. Some forms and scopes of justice have not been examined in the existing AMR ethics literature—for instance, more-than-human, Ubuntu, *vincularidad*, Confucian, and Buddhist justice. Are these, too, needed for a holistic evaluation of whether a measure might fall within a just transition, and what justice requires for a just transition? To work towards answering this question, we present two case studies, each of a particular measure implemented in a particular context that intuitively seems to form part of an *unjust* transition. By highlighting issues of justice raised by the cases, we demonstrate how some of these lie outside the traditionally considered forms of distributive, procedural, and structural justice from the AMR ethics literature, and outside the scope of traditional forms of justice included in the idea of a just transition, including distributive, procedural, and restorative justice. Our analysis demonstrates why and which forms and what scope of justice are appropriately added to our multifaceted concept of a just transition.

In our first case study, we explore how the enforcement of laws banning on OTC access to antimicrobials aims to strengthen the associated regulatory system to achieve greater control and compliance with regulations on access to antimicrobials. The policies are contained in the Kenyan National Policy (KNP) on Prevention and Containment of AMR and its corresponding NAPs for the periods of 2017–22 and 2023–27. In our first case study, we look at how they may inadvertently perpetuate injustices for particular populations in contexts such as Kibera—Kenya’s largest informal settlement, characterised by slum conditions. We explore this as not only a form of distributive injustice but also structural injustice and Ubuntu injustice.

Our second case study looks at a traditionally pastoralist Maasai community in rural southern Kenya, and their experiences of stewardship measures corresponding to the NAP guidelines, specifically the legal requirement for antimicrobial prescriptions for livestock, and the guidelines on maintaining withdrawal periods after animal illness and antimicrobial treatment. This case highlights the roles of more-than-human justice and epistemic justice. The two case studies are particularly relevant to the lived experiences of populations, as 70% of Kenyans live in rural communities, and among the urban population areas, more than half (51%) live in informal settlements.^32^ These data mirror the broader sub-Saharan African (SSA) urban landscape.^33^ Furthermore, given Charani et al.’s recent evidence synthesis that found similarities between NAPs across the region, the challenges highlighted by our case studies may be echoed elsewhere, as well as the conclusions we draw regarding the demonstrable need for multifaceted approaches to justice when securing a just transition for AMR.^34^

The SSA region is facing challenges associated with the dual burdens of infectious and non-communicable diseases. It is home to the highest infectious disease burden of any region, including an ongoing HIV/AIDS epidemic, with emerging challenges in resistance to antiretrovirals, antimalarials, and increasing incidence of non-communicable diseases such as cancer.^35^ Addressing the dual burdens is in part reliant on the efficacy of antimicrobials, and resistance is both an increasingly acute threat and a reality. SSA currently has the highest mortality rate attributable to AMR, although the burden specific to, for example, antibiotic resistance is forecast to shift to rest primarily on South Asia, Latin America, and the Caribbean in the coming decades.^36^ Across the SSA region, studies show worrying trends. These include data on antibiotic resistant bacteria being found in newborns, with possible associations with overuse of antimicrobials in agriculture.^37^ There are also indications of children who are newly diagnosed with HIV that is already resistant to antivirals.^38^ The case for surveillance, stewardship, and controls to preserve the efficacy of antimicrobials across SSA is strong. Stronger still is the challenge to develop just policies that will not negatively impact the most vulnerable in a region where socioeconomics, culture, politics, poverty, and precarity in everyday work are reported to be entangled with use of antibiotics.^39^

The Kenyan government has adopted several strategies to tackle AMR across communities, chief among them is the development of the NAP, which has been “identified as a positive outlier [for] demonstrating a comprehensive, multi-sectoral, and stakeholder-led approach.”^40^ The framework for its delivery, monitoring, and evaluation mirrors the Kenyan decentralised political system, operating both at the national and county levels using a One Health, cross-sectoral approach. Understanding both barriers to NAP implementation and the possible negative externalities of successful implementation requires an approach that is historically situated, which we turn to now.

### 4.1 Case Study 1: Access to antimicrobials over-the-counter among Kibera residents

Kenya is a former British colony that gained independence in 1963.^41^ Archetypally of former colonies, it inherited an enclave economy, marked by dual economic structures where a relatively modern formal economy serving the minority colonial population can run in parallel to a differently constructed agrarian economy to serve the majority of the population.^42^ In today’s post-colonial Kenya, wealth is concentrated in neighbouring metropoles Nairobi and Kiambu, which are the largest contributors to the country’s GDP. These areas are marked by large wealth disparities. Nairobi, specifically, is marked by economic inequality that has an impact on the epidemiology of infectious and chronic disease, with varying distribution and associated risk across geographies, demography, and class.^43^ Nairobi also faces the challenge of inequity in access to healthcare services, with a bearing on patterns of antimicrobial use among various populations.^44^ For some of the population living in slums, colonial history as a macrosocial process remains relevant and is associated with the epidemiological landscape and challenges in creating just responses to tackling AMR. For example, Kenya’s largest informal settlement, Kibera, has a history “rooted in the segregation of colonial rule; it is sustained by the continuing injustice of land policies and the multiple complications involved with upgrading urban settlements.”^45^

Nubians arrived in Kenya over a century ago, and after that, they were forcibly transported from Sudan as part of the British colonial army. In an act of defiance, they abstained from fighting in the uprising, with great implications that have reverberated across generations; the British denied them title deeds, and from the Kenyans, they failed to gain recognition or favour.^46^ Across five generations, this has manifested in discriminatory policies towards the Nubian community. Reflecting on her father’s life, Kenyan Nubian writer Waziri remarks: “It is so absurd, to the extent that without an ID, one cannot legally die, which is what happened to my father. He does not have a death certificate because he did not have an ID. The state neither recognized his life nor his death—he never existed.”^47^ Nubians have endured a long and complex journey towards being recognised by the state and obtaining corresponding legal documentation such as national ID cards issued to all Kenyan citizens. These are necessary for navigating everyday life in Kenya—for voting, education, employment, and home buying, as well as accessing healthcare, whether registering for health insurance or accessing treatment in public hospitals.^48^ Whilst there have been recent shifts in the requirements to qualify for a national ID card, there are people who continue to have great difficulty in proving that they qualify for citizenship because they cannot produce required documents, such as parental ID cards, which would be impossible for people like Waziri, whose fathers “never existed.”^49^

Today, Kibera has mushroomed into Kenya’s largest informal settlement and the largest urban informal settlement on the African continent. It is characterised by challenges that are typical of slums such as the absence of public services.^50^ In Kibera, where challenges in access to water, sanitation, hygiene, and wastewater management persist and give rise to unhygienic conditions that promote rapid spread of pathogens, there are reported high levels of antibiotic resistance in stool samples collected in households.^51^ Whilst Nubians now account for a small part of the Kibera population and indeed the Kenyan population, the ways that they are treated and limited in their healthcare interactions due to the legacy of injustices during colonial times and their perpetuation through governance structures today highlights the need for forms of justice that can account for their experiences and point towards solutions for a just transition that does not marginalise the Nubians and others like them and their ways of accessing antimicrobials.

In Kenya, drug access and trade are regulated by several laws, including the Pharmacy and Poisons Act, which provides the legal framework from which NAP guidelines are derived. It regulate the necessity of a prescription for the dispensation, sale, and provision of antimicrobials to individuals.^52^ Whilst the laws and regulations are clear on the legal conditions under which antimicrobials are to be used, Kenya’s NAP recognises the common occurrence of OTC sales across its human and animal health sectors; thus, the NAP sets out to review and strengthen the laws as a strategic objective for the period of 2023–27. The Kenyan NAP particularly recognises the issue of OTC access to antimicrobials in informal settlements, and notes mixed accounts of how prevalent the practice of selling antimicrobials without a prescription is among pharmacies in and surrounding Nairobi.^53^ The state aims to reduce OTC access, which intersects with the challenges faced by Nubians living in Kibera, in which they are particularly vulnerable to injustices associated with areas of action such as strengthening enforcement on the OTC ban as part of the NAP. Due to health system challenges and lack of access to healthcare and health insurance—getting a prescription for an antibiotic, for example—is relatively more difficult for Kibera residents, and more so for Nubians without national IDs in particular. Mukokinya et al. reported a low level of off-prescription antibiotic dispensation among pharmacies in Nairobi, with 94.1% of antibiotics being dispensed with a valid prescription.^54^ Reducing OTC access can provide benefits in reducing misuse and overuse. However, it may very well push populations out of the regulated drug market and into the unauthorised market out of necessity to access anti-microbials in contexts where getting a prescription is especially difficult, if not impossible. This makes those populations more liable to suffer the negative consequences that are commonplace in the unregulated market such as exposure to harmful or ineffective falsified medicines. Negative consequences include failure to treat conditions, and the potential for consuming products contaminated with toxic substances.^55^ There are some strong arguments, then, for shoring up enforcement in order to, for example, decrease antimicrobial sales off-prescription. However, these arguments are not without negative externalities such as those mentioned above, that may not only compound injustice through exposing individuals to risk, but also compounds AMR, as substandard drugs can contain subtherapeutic levels of the active drug component, which can drive AMR.^56^ Our concerns are, therefore, associated with the measures of success, specific to OTC sales. For example, a decrease in OTC sales may represent a decrease in *necessary* antimicrobial use by certain groups such as those in the Nubian community, who are still waiting for legal documentation and have limited means of accessing treatment. The concerns this raises are threefold: first, broad-brush rather than nuanced enforcement will undermine the government’s goal “to ensure, for as long as possible, continuity of successful treatment and prevention of infectious diseases with effective and safe medicines that are quality-assured, used in a responsible way, and accessible to all who need them” as outlined in the first NAP.^57^ Second, it places a greater risk of inaccess on historically marginalised communities in terms of inaccess to antimicrobials when they are needed if an OTC ban is successfully enforced, and a greater risk of receiving falsified or substandard medicines is still placed on the same population. None of these issues are adequately accounted for across the KNP and NAPs, nor is the fact that these issues compound legacies of harm for marginalised populations. Whilst the KNP and NAPs have recognised drivers of AMR associated with health system challenges, there are no proposed strategies that address vulnerable populations such as the Nubians. Instead, the focus on strengthening enforcement without effectively addressing the underlying health system and socioeconomic challenges that drive OTC access will further exacerbate inequitable access to healthcare, in a particularly problematic way that builds on a legacy of historical exclusion of those living in informal settlements and, for the Nubians, a history of oppression—colonial and otherwise.

At this point in the case study, potential injustices raised by the enforcement of an OTC ban on the residents of Kibera, and Nubians in particular, centre on the structures that make Nubians more vulnerable to policy-related harms than other groups. Structured injustice does not stem from, or usually apply only to, individuals; rather, it affects groups and results from the production and perpetuation of institutions that oppress groups. In parallel, structural injustice arises from impersonal processes that create disadvantages for particular populations.^58^ Racial and gender disparities are examples, as is the continuation of institutions and systems that perpetuate colonial legacies. The barriers to accessing the healthcare system that Nubians experience are representative of structured and structural injustice. Furthermore, insofar as the Kenyan NAPs exacerbate barriers to healthcare by cutting off informal routes to accessing drugs without prescription that Nubians have relied on in the past, it also represents a structured injustice. Whilst it is important to ensure that antimicrobials are not being used in ways that unnecessarily risk worsening AMR, this must be done with an eye on avoiding perpetuating structural and structured injustice for populations such as the Nubian community in Kibera, particularly those awaiting legal recognition and documentation. Other supports *must* therefore be put in place for them. For instance, promoting structured justice may involve accompanying the NAP with further supports for seeking healthcare for those without national IDs, implementing health supports or improving water, sanitation and hygiene measures to prevent the spread of infections in informal settlements, and dismantling the processes that create barriers to getting national IDs for Nubians, such as the requirement for showing parental ID cards to qualify for citizenship. These are all actions that should be considered in the implementation of the Kenyan NAP to avoid exacerbating structured injustice for.

The above is a specific case that highlights a broader challenge—the failure to include populations on society’s margins in our approaches to tackling AMR. This will undermine efforts to tackle AMR. For example, the WHO underscores the importance of just access to antimicrobials among refugee and migrant populations and the challenge of legal status as a foundational determinant of health.^59^ It effectively highlights how systemic and impersonal barriers, such as unaffordable healthcare, can redirect help-seeking away from authorised health providers and towards unauthorised pharmacies and informal markets, with implications for health authorities’ capacities to monitor AMR.

A second type of injustice raised by Case Study 1 relates to Ubuntu philosophy, best understood through the Xhosa maxim, “Ubuntu ungamuntu ngabanye abantu.”^60^ That translates to “a person is a person through other people.” It forms the basis of ethical traditions among the Nguni people of southern Africa, and has come to be considered representative of relational ethics across Africa, resonating with philosophies across many areas of the continent.^61^ It relates people not as individuals, but as part of groups and communities, and as a form of justice, it has been applied to issues such as rehabilitating criminal offenders back into communities, reconciliation after apartheid in South Africa, and sustainable development and use of resources that involve communities as resource stewards.^62^ In the context of the KNP, NAPs, and subsequent enforcement of the ban on OTC salesthere are shortcomings in fulfilling Ubuntu justice as a form of justice. These are associated with the failure to recognise members of the Nubian community as part of the broader Kenyan community, alongside the systematic denial of their legal personhood. This curtails the Nubian community’s access to important ways of being a part of Kenyan society by relegating them to legal non-personhood, and it imposes greater health risks on them as a result of inaccess to healthcare. Distributive justice might allow us to consider the distribution of health- and wellbeing-related burdens and benefits among individuals, to determine whether this policy fulfils the requirements of justice. Ubuntu justice can offer more: it focuses on the effects of the policy not only on individuals but also on relationships, interactions, and togetherness, at both micro- and macro-levels. On a small scale, stronger enforcement of the OTC ban, along with an effective ban of illegal and unauthorised drug shops in Kibera, separates the Nubians without national IDs from others in the informal settlement who do have access to national IDs, and therefore greater access to antibiotic prescriptions.^63^ These may create pronounced differences between sub-communities with and without national IDs in terms of health inequalities. On a larger scale, all residents of Kibera may face Ubuntu injustice insofar as their membership of Kenyan society and their vulnerability to worse health effects as a result of the effective enforcement of the ban on OTC sales in a context where policy has left them behind and failed to include them in the “we” that determines policy that will serve Kenyans present and future, but rather are excluded from consideration.

### 4.2 Case Study 2: Livestock farming and animal antibiotic use among Maasai

Whilst the urban population across much of SSA has been expanding, the majority of the SSA population continues to reside in rural areas. This is true for Kenya, which boasts a large and diverse rural population. The various rural populations must not be treated as a monolith—they have different histories, geographies, practices, cultures, and values. We therefore centre pastoralist communities in southern Kenya specifically in this case study, drawing on examples from the Maasai people who typically live in southern Kenya and Tanzania. Pastoralists are traditionally nomadic people whose animal husbandry practices for live-stock production are reliant on traditional knowledge systems.^64^ For the Maasai, livestock plays an important role in maintaining the fabric of social life, with both economic and cultural significance. For example, livestock is an important aspect of accessing the institution of marriage within the community, where polygamy remains commonplace and a bride price is required, often in the form of livestock.^65^ Children are introduced to looking after livestock at an early age, initially looking after kids and calves around the compound from as early as five years old, with boys progressing to herding goats, sheep, and cattle that are taken outside the family compound for grazing during the day and brought back at dusk.^66^ Traditional practices in raising livestock are rooted in long-tested experiences, based on inference and adaptive practices that are shaped around tracking and grazing for food and water across large arid/semi-arid landscapes for livestock maintenance, production, and sustenance of livelihoods.^67^ We do not wish to imply that all of the cultural practices listed above that are affected by changes in access to antibiotics for livestock are valuable, and therefore raise issues of justice. However, gaining a comprehensive understanding of the context in which livestock farming plays a role is essential for evaluating the impact of changing antibiotic use and access on aspects of pastoralists’ lives and whether these do, indeed, cause injustices or not.

The Maasai pastoralists’ knowledge and practice occur in a landscape that is, at least theoretically, governed by national and international norms concerning veterinary antibiotic use. The Kenyan NAP notes that whilst the current AMR burden in livestock is unknown, there is evidence of poor practice in infection prevention, exacerbated by off-label and illegal use of veterinary antibiotics as growth stimulants. This is despite existing laws, including the Pharmacy and Poisons Act discussed in Case Study 1, that forbid access to veterinary antibiotics without a prescription from animal health professionals.

In a paper titled “We are doctors,” Mangesho et al. describe antibiotic use for livestock protection among the Maasai. Reflecting the previously established high rates of drug administration without input from external animal health professionals, the study found self-reported high confidence in how livestock were treated against a backdrop of low confidence in animal health service providers.^68^ This was linked to culturally ingrained processes through which Maasai develop skills in maintaining animal health and welfare, and see themselves as experts on animal health and welfare, both as a result of their own experience and as part of a cultural inheritance. This perception is underscored by the frequent lack of access to veterinarians and other animal health professionals near Maasai communities.^69^ Linking knowledge with behaviour, one study holds that administration of antibiotics by Maasai herders directly to animals is as high as 90%, with little input from external animal health professionals.^70^ Selling and consuming animal products without observing periods of withdrawal after treatment was also reported as common.^71^ Conformance with antimicrobial stewardship measures would require disposal of milk/meat products produced within withdrawal periods to reduce the risk of antibiotic residues in products for human consumption. Withdrawal periods can be understood as the necessary or recommended intervals between administration of a drug to an animal for treatment and the time at which the antibiotic residue levels in the animal’s meat or milk fall below the maximum threshold, making the meat and milk safe enough for consumption with a notable decrease in potential transmission of resistant bacteria from animal products to humans. Yet, Maasai perceptions of the cultural importance of livestock can have an impact on perceptions of external animal health providers and the validity of taking advice from health providers on interventions like withdrawal periods, which can be in violation of Maasai social norms. Given the importance of cattle as gifts from God and their social and economic importance to the Maasai, any required disposal of products that have not met withdrawal requirements poses a direct challenge to cultural survival.^72^ Many community members report never having seen anyone falling ill from the consumption of animal products produced without compliance with a withdrawal period, or otherwise.^73^

There are two potential injustices highlighted in the case study. The first is distributive, and concerns whether the burdens of compliance with stewardship measures are fairly distributed where they primarily befall a population that will face greater health risks and financial risks from stewardship measures than other populations whose uses of antibiotics may also drive AMR. These concerns have been covered more thoroughly in the existing AMR literature, and we just note them here.^74^ The second form is epistemic injustice. We can identify violations of two forms of epistemic justice—testimonial justice and hermeneutical justice—in the imposition of requirements for seeking veterinary advice, in particular.^75^ A violation of testimonial justice occurs where the “ethno-veterinary” knowledge and practices of the Maasai are undervalued due to prejudice against them and the assumption that their knowledge of livestock farming is less legitimate than that produced by commercial farmers, veterinarians trained in Western veterinary medicine, or other non-marginalised groups, without adequate justification. As some studies indicate, certain practices conducted by the Maasai are set in a context of great pride and understanding about pastoral history, specific approaches to disease surveillance and identification, and appropriate treatment.^76^ Yet, even among the studies we have examined that discuss the importance of Maasai practices, the Maasai are often implicitly treated by the authors in the language (“ethnoveterinary practices,” non-equation with animal health professionals, etc.) used to indicate less knowledge or less relevant knowledge than veterinarians and animal health professionals.^77^ We might ask ourselves whether the same marginalisation would occur for generationally passed-down knowledge on farming practices among farmers in high-income countries, or whether they might be treated as having relevant knowledge of animal welfare. If there is a discrepancy, why? Is it adequately justified? We do not seek to answer such questions here, but we believe they require thorough investigation, using concepts of justice like testimonial justice. For instance, by learning more from the Maasai about how their practices of allowing animals to roam around family compounds arise, we learn more about disease surveillance. From understanding how observations of pastoralist-administered treatment occur, we can learn more about the embarrassment that the Maasai associate with being unable to identify relevant symptoms of disease in animals and an appropriate treatment strategy. We can explore whether such embarrassment is the reason why antibiotics might be used after alternative potential treatments fail. A second form of epistemic injustice, hermeneutic injustice, occurs when the Maasai people are not given the same tools (language and other interpretive resources) that are needed to make sense of and convey their experience. For instance, why is it an “embarrassment” not to be able to identify symptoms of disease, and does this indicate respect for epidemiological expertise that goes uninterpreted? Whilst these kinds of knowledge and practices may not always align with recommended antibiotic use, it is important to understand them as a baseline for integrating traditional practice with stewardship measures.

To address epistemic injustices effectively requires engaging with traditional animal health practices in earnest alongside foregrounding the relevant “voices and ways of knowing.”^78^ One study has suggested that by drawing on pastoralists’ knowledge, instead of contradicting it, more can be done to motivate behaviour change in areas where it is needed—for instance, in supporting the observation of links between antimicrobial use and subsequent increased rates of treatment failure, and/or by leveraging sources of information the Maasai use including agro-vet employees (company staff providing agricultural services and limited prescription veterinary services)–instead of simply promoting the knowledge of the often inaccessible and hegemonic veterinary professionals who are currently required to provide prescriptions.^79^ Nomenclature has played a key role in advancing colonial ideology and epistemic erasure.^80^ The imposition of concepts manufactured elsewhere can undermine and infantilise indigenous knowledge. Without opportunities to both learn about and communicate to others about the needs of their animals and the trade-offs they face when asked to curb antibiotic use, the Maasai are denied hermeneutical justice.

Without understanding the knowledge foundations of Maasai pastoralist practices, injustice and ineffectiveness in AMR interventions may result. On the other hand, we might promote justice and effective intervention by aiming to provide spaces for developing their own language and learning around AMR among pastoralists. For example, around 50% of pastoralists in one study reported observing an increase in treatment failures when they started farming, and were concerned with reforming farming practices (though causes of treatment failure were not documented).^81^ Why not integrate this knowledge and motivation for change into policymaking and alter communication efforts to address this AMR-related priority identified by the pastoralists themselves? This may be difficult to achieve in the way current antimicrobial stewardship policies are set out and applied to pastoralists in southern Kenya. We do not aim to suggest here an approach that overvalues Maasai knowledge or ignores the role of veterinary medical expertise in determining appropriate practices like prophylactic uses of antibiotics and appropriate withdrawal periods. What we want to suggest is that the requirements of epistemic justice should shift our aims towards developing a mutual understanding between the types of knowledge that are relevant to pastoralist practices in southern Kenya.

Current policies also have adverse implications for leveraging traditional healthcare practices in African public health. To that end, we suggest embedding decolonial scholarship in AMR-specific public health education as an approach to bridging the gap between traditional ways of being and knowing, the increasing use of western medicine, and the challenge of AMR with the view to increase efficacy of public health education, as well as contribute to the de-silencing of meaningful discourse that has the potential to contribute to effectively tackling challenges such as AMR within these communities. This would be in keeping with calls for holistic approaches to addressing AMR that factor in sociocultural and belief systems.^82^

A further consideration in Case Study 2 is that whilst the Maasai across Kenya and Tanzania continue to maintain traditionally pastoralist lifestyles, the last few decades have seen an increase in economic diversification across these communities. This has enabled a shift from purely subsistence farming practices towards incorporation into national economies, through paid work and entrepreneurial projects in various sectors such as tourism and wildlife conservation.^83^ Maasai pastoralists historically herd their livestock in areas that are inhabited by wildlife, with some wildlife–livestock–human interactions at watering and grazing areas. This has increased since the establishment of wildlife conservancies that are deemed as sustainable interventions for protecting wildlife and their ecosystems. For example, in southern Kenya, conservancies have revived the lion population through the implementation of programmes that pay Maasai community landowners for the lease of designated conservancies.^84^ While these have been touted as sustainable interventions for conservation, new sets of challenges have emerged from them, including increased competition for resources such as water and more wildlife–livestock–human interaction. This has implications regarding increased risk of bacterial transmission and higher levels of risk of enteric disease for both animals and people.^85^ It also raises questions about the appropriate scope of justice, and whether, aligning with a more-than-human approach, the animals affected by changing interaction with humans, changing antimicrobial use, and increases in conservancies ought also be considered as subjects of justice. One indication that animals may already be seen as subjects of justice by the Maasai people comes from their support for using land for wildlife conservation alongside farming practices based on the concept of “nashulai.”^86^ This means “a place of harmony where community and wildlife live in balance and mutual benefit, where the spirit of the people and the spirit of nature and wildlife come together into a common song.”^87^ This raises interesting questions about the intersections between wild animals, livestock, humans, and the environment when considering the scope of applying justice considerations.

Indigenous people have long understood the oneness of people, herd animals, wildlife, and place that the Westernised concept of One Health is geared towards. Long before the introduction of One Health, there has been an understanding of relationality and totality of all living things, that is shaped by a dependence on each other. This is embedded in traditional belief systems that denote the responsibility to look after land and animals.^88^ For the Maasai pastoralists, for example, there exist opportunities to leverage “nashulai” and explore the balance between wildlife, nature, and people. In this way, there is scope to move beyond translation and grafting on new terms, such as One Health, and instead move towards tapping into local knowledge systems, as part of fulfilling the requirements of epistemic justice. This, too, highlights the role of decolonial scholarship in developing understandings of the relevant facets and conceptions of justice in a just transition. By only importing the concept of One Health—or indeed related universalised concepts such as more-than-human justice—instead of also working to understand the role of concepts developed in and by a particular place and people like “nashulai,” we not only introduce epistemic harms and perpetuate epistemic colonialism, but also fail to recognise specific elements that do not translate across seemingly related concepts such as One Health.^89^ We may, for example, miss references to ecosystem stewardship, links between land prosperity and human prosperity, and the importance of intergenerational passing down of knowledge on sustainable living with wildlife and livestock. Fair treatment might mean something different in this context than another, particularly where justice is broadened out to the consideration of other beings than humans.

The issues raised in relation to Case Study 2 highlight the importance of multiple forms of justice, including distributive and epistemic justice, alongside a broader scope of justice through a more-than-human approach. What is essential is that morally relevant concepts and knowledge such as “nashulai”, in this setting, are used where possible. The case studies also underscore the importance of nuanced and ongoing discussions between communities and policymakers—discussions that are responsive to both the heterogeneity of rural populations and the evolving practices of specific populations, not only in relation to antimicrobial use but also in relation to new developments in tourism, wildlife conservancy, environmental protection, and so forth.

## 5 Conclusion: what a just transition for AMR requires

In this paper, we have outlined what forms of justice are traditionally considered in the AMR ethics literature and why these are overly narrow. We have noted the forms and scope of justice traditionally included in the concept of a just transition and why these are too narrow. The case studies we have presented offer an opportunity to explore injustices that can result from the transition towards a different AMR future, through stewardship measures contained in NAPs. These have diverse implications for different communities, and whilst they may fulfil the needs of some, they clearly do not fulfil the needs of all. A broadening of both the forms and the scope of justice considered within a just transition is required if we are to address the injustices identified in these case studies. We call for further work to be done to explore what a multifaceted, broad-scoped conception of what a just transition for AMR could entail for particular future interventions.

## Footnotes

1 Khadse, Ugemuge, and Singh 2023.

2 Littmann, Viens, and Silva 2020.

3 UNDP Climate Promise 2022.

4 Coninx et al. 2022; Varadan et al. 2024.

5 Coninx et al. 2022; Varadan et al. 2024.

6 Zimm et al. 2024.

7 World Health Organization 2022.

8 UNDP Climate Promise 2022.

9 Wang and Lo 2021.

10 Coninx et al. 2022.

11 Wang and Lo 2021.

12 Varadan et al. 2024.

13 UN Committee on Economic, Social and Cultural Rights 2000.

14 Collins et al. 2018.

15 Lu 2017. See Lu’s discussion for the distinction between “structured” (institutionally mediated) and “structural” (resulting from informal, impersonal systems and processes) injustices.

16 Vikesland et al. 2019.

17 World Health Organization 2023.

18 Charani et al. 2023; Littmann 2014; Reid 2020. However, see Tegama 2023 for emerging work in the area. Compare Ubuntu justice with existing Ubuntu philosophical approaches to other issues like euthanasia, for example, in Lougheed 2025. Compare *vincularidad* with existing applications of the *vincularidad* concept to the ethics of zoonotic spillover events, for example, in Rodriguez 2024. Compare Confucian justice to emerging applications of *yi* from Confucianism to global health and health equity implications, in Jecker and Chung 2025. Compare Buddhist justice to emerging Buddhist approaches to issues such as euthanasia; see, for example, McCormick 2013.

19 Compare this with existing approaches to multi-species justice in environmental politics, for example, in Celermajer et al. 2020.

20 Littmann and Viens 2015.

21 Giubilini 2019; Littmann 2014; Schwenkenbecher 2020; Selgelid 2007.

22 Charani et al. 2023; World Health Organization 2015.

23 Poomchaichote et al. 2024; Schwenkenbecher 2020.

24 Fricker 2007, 1.

25 Nijsingh and van Bergen 2019; Tegama 2023.

26 Tegama 2023.

27 Young 2011, 52.

28 Krockow and Tarrant 2019; Merrett et al. 2016.

29 Johnson 2024a, 2024b.

30 Reid 2020.

31 Reid 2020, 268.

32 World Bank 2023.

33 Ibid.

34 Charani et al. 2023.

35 Ishengoma et al. 2024; Mpundu and Ali 2020; Omotoso et al. 2023.

36 GBD 2021 Antimicrobial Resistance Collaborators 2024; Murray et al. 2022.

37 Portal et al. 2024.

38 Anderson et al. 2024.

39 Nabirye et al. 2021.

40 Charani et al. 2023, 468.

41 Dill and Crow 2014.

42 Kanyenze et al. 2011.

43 Omulo et al. 2021.

44 Ilinca et al. 2019.

45 Dill and Crow 2014, 187.

46 Akcay 2014.

47 Waziri 2019.

48 Nasubo and Ngira 2024.

49 Waziri 2019.

50 Wamukoya et al. 2020.

51 Omulo et al. 2021.

52 Pharmacy and Poisons Act: Revised 2012 [1989] n.d.

53 World Health Organization 2022.

54 Mukokinya et al. 2018.

55 Gulumbe and Adesola 2023.

56 Ibid.

57 Government of Kenya 2017.

58 Kapelner 2021.

59 World Health Organization 2021.

60 Battle 2009, 3.

61 Mangena n.d.

62 Nnodim and Okigbo 2024; Oko Elechi, Morris, and Schauer 2010.

63 Jacobs and Richtel 2019.

64 McCabe, Leslie, and Deluca 2010.

65 Coast 2006.

66 Ibid.

67 Hussein, Abdi, and Ahad 2023; Scialabba 2022.

68 Mangesho et al. 2021.

69 Caudell et al. 2017.

70 Ibid.

71 Hussein et al. 2023.

72 Mangesho et al. 2021.

73 Ibid.

74 Littmann and Viens 2015.

75 Fricker 2007.

76 Caudell et al. 2017.

77 Caudell et al. 2017; Mangesho et al. 2021.

78 Tamale 2020, 176.

79 Caudell et al. 2017.

80 Ndlovu-Gathseni 2018.

81 Caudell et al. 2017.

82 Asiimwe et al. 2021; Leadingham et al. 2019.

83 Coast 2006; Mangesho et al. 2021.

84 Mangesho et al. 2021.

85 Bett et al. 2024.

86 Nashulai Maasai Conservancy, n.d.

87 Weston 2025.

88 Murove 2009.

89 Johnson et al. 2024; Tschakert 2020; Winter 2022; Zhao, Popkewitz, and Autio 2022.
